# Assessing the Reach and Engagement Effectiveness of Disseminating Food and Nutrition Information on Social Media Channels

**DOI:** 10.3390/foods13162535

**Published:** 2024-08-14

**Authors:** Daniela C. Avelino, Carolyn A. Lin, Molly E. Waring, Anna J. Barbosa, Valerie B. Duffy

**Affiliations:** 1 Department of Allied Health Sciences, University of Connecticut, Storrs, CT 06269, USA; daniela_carolina.avelino@uconn.edu (D.C.A.); molly.waring@uconn.edu (M.E.W.); anna.barbosa@uconn.edu (A.J.B.); valerie.duffy@uconn.edu (V.B.D.); 2 Department of Communication, University of Connecticut, Storrs, CT 06269, USA

**Keywords:** social media channels, food and nutrition information dissemination, reach and engagement effectiveness, social media uses and gratifications, hedonic vs. utilitarian value, Facebook, Instagram

## Abstract

This study utilized Facebook and Instagram as communication channels for disseminating evidence-based food and nutrition information to low-income adults. From February 2021 to October 2022, 442 identical posts were shared across both platforms for audience reach and engagement. Posts were categorized in two ways: hedonic and three levels of utilitarian (informative, convenience, utility), based on widely applied social media uses and effects theory (Uses and Gratifications Perspective); and food/nutrition topics (dietary guidance, mealtime behaviors, recipes, food resource management, health behaviors, and community building). From predominantly image-based posts (82.6%), reach and engagement for Instagram (136,621 versus 6096, respectively) outperformed Facebook (83,275 versus 1276, respectively). Analysis of covariance of rank-order reach and engagement metrics (likes, replies, shares) showed Facebook engagement was consistent across hedonic and utilitarian categories while Instagram showed highest reach and engagement for utilitarian posts, especially those emphasizing food affordability. Facebook and Instagram differed in which food/nutrition topics achieved maximal reach and engagement. Fifteen posts were randomly selected for qualitative analysis to identify features reflecting engagement levels. Low-engagement posts featured low-color-contrast or less-appealing images, especially on Instagram. This study offers insights for practitioners and researchers aiming to use social media to promote healthy food and nutrition.

## 1. Introduction

Food- and nutrition-related information has grown in popularity on social media platforms, with frequent posting by health organizations, individual users, and food and wellness influencers [1]. Unfortunately, most individuals from 2 to 60+ years old in the U.S. fail to consume a healthy diet promoted [2,3] by the Dietary Guidelines for Americans (DGA) [4]. Moreover, low-income adults are more likely to have inadequate dietary intake and be at increased risk for chronic disease (e.g., obesity and heart disease) across their lifespan [5]. Technology-based strategies can improve access to affordable healthy foods by providing education, facilitating discounts, offering convenient shopping options, and fostering community support for low-income families [6,7,8,9]. Previous research has found that participants in food assistance programs are interested in nutrition information that is shared via social media [10].

Social media could be an amplifier for disseminating evidence-based food and nutrition information that promotes favorable health outcomes aimed at low-income individuals [11]. It cannot promote social devaluation and isolation, as recently reported in adolescents from low-income families [12], and instead promotes engaging and evidence-based information that is responsive to local needs to promote healthier eating [13]. There is growing evidence that social connections have a positive impact on improving health outcomes [14,15]. Guidance on evidence-based and relevant nutrition education is imperative for effectively engaging local communities through social media channels [16].

The Supplemental Nutrition Assistance Program Education (SNAP-Ed) is a federally funded grant program in the U.S. [17] that supports evidence-based nutrition education interventions and policy/systems/environmental changes for low-income individuals to facilitate choosing and accessing foods aligned with the DGA, food resource management, food safety, diet quality, obesity prevention, and nutrition security [18]. Studies have analyzed Facebook as an intervention for low-income participants and found increased engagement in obesity prevention (e.g., views, likes, and comments) [6] and healthy eating on a budget curriculum [8]. Although social media is a promising modality for delivering nutrition education to low-income audiences [6,7,8], empirical research and extension efforts are lacking [19]. Thus, the present study aims to describe the overall consumer reach and engagement of an online SNAP-Ed program implemented on social media platforms. Specifically, this study aligns with a theory-guided framework, which connects program activities with social–psychological theoretical concepts for a comprehensive evaluation to inform program enhancement [20]. Our theory-guided framework describes the perceived affective and cognitive user gratifications derived from the hedonic and utilitarian values that are associated with food and nutrition information consumption.

Food companies strategically use social media to target consumers and their eating habits [21]. By comparison, nutrition and health educators have yet to fully gain traction on this online communication channel to positively influence the attitudes and behaviors of their target audience [22]. Theory-guided frameworks can help improve understanding of consumer motivations for engaging in food- and nutrition-related content in the media (e.g., cooking shows), including social media. In mediated communication research, the most used theory to explain why consumers utilize social media is the Uses and Gratifications (U&G) theory [23], which asserts that people are self-motivated active agents who select and use media content to fulfill their own psychological needs. The U&G theory includes dimensions for ritualistic and instrumental motivations [24,25] that are respectively parallel to hedonic and utilitarian values or motivations from a consumer behavior perspective. As suggested by Lin and Rauschnabel [26], audiences driven by ritualistic or hedonic motivations will use social media platforms to meet their affective and enjoyment-oriented needs such as entertainment, diversion, escape, and relaxation. In contrast, those prompted by instrumental or utilitarian motivations will choose social media content to meet their cognitive- and functionality-oriented needs such as keeping up with the news, learning new skills, sharing information, and developing social connections [25,27,28].

The hedonic versus utilitarian motivation theory has also been applied to consumer decision-making in food choice in relation to its nutritional value. Specifically, past research has conceptualized hedonic value as the affective motivational dimension that drives individuals to seek and experience the enjoyment of food consumption [29]. In essence, the basis of hedonistic consumption is to offer an enjoyable, pleasurable, happy, or amusing eating experience [30]. To wit, the top drivers of what people chose and purchase to eat are the hedonistic reason/motivation of “taste” [31] or liking the sensory component of food and beverages, followed by utilitarian reasons/motivations of price, healthfulness, convenience, and sustainability. Physical exercise could also be hedonistically pleasurable, making people feel good and have fun as well [32]. Individuals who regularly exercise are more likely to follow healthy lifestyles and eating habits [32]. Likewise, prior work has operationalized utilitarian value as the cognitive motivational dimension that guides individuals to search for and learn about the nutritional value and health benefits of food [33]. Utilitarian eating values are generally concerned with convenience, price, nutrition, and other health-related aspects [34,35]; these are consistent with existing research on food consumption and the main factors that can influence food choices [31,36,37]. A recent qualitative study on the gratifications of using food media found that participants used food content to make informed grocery choices, such as shopping online and purchasing cheaper or more sustainable food [38]. Summarizing the application of hedonic versus utilitarian values as motivations to explain consumer attitudes toward food and nutrition, Voss et al. suggest that hedonic value is related to seeking an enjoyable or pleasurable eating experience—and the utilitarian value is affiliated with receiving the perceived benefits of healthy meals, such as their nutritional value and other health-related components [39]. 

This is an exploratory study regarding the effectiveness of utilizing two different social media platforms—Facebook and Instagram—to reach and engage low-income individuals with nutrition education to support healthier diets and access to healthy food. This study had the following aims:

1. To describe the social media reach and engagement frequency and trends generated from Facebook and Instagram between February 2021 and October 2022.
2. To classify and describe the social media reach and engagement frequency based on user gratifications (as reflected in hedonic versus utilitarian motivation) and nutrition education categories.
3. To conduct a qualitative analysis of high-, moderate-, and low-engagement social media posts to identify key characteristics that contribute to consumer engagement.

## 2. Materials and Methods

### 2.1. Characteristics of the Social Media Channels

This study evaluated the reach and engagement of posts created by the *Healthy Family Connecticut* (HFCT) nutrition education team for their public Facebook and Instagram accounts. The SNAP-Ed team at the University of Connecticut, College of Agriculture, Heath, and Natural Resources, administers the accounts (https://healthyfamilyct.cahnr.uconn.edu/; accessed on 10 August 2024). HFCT was created in February 2021 to deliver evidence-based SNAP-Ed to the public. The social media outreach and engagement strategy was created from an intervention mapping exercise, which entailed a needs assessment including systemic reviews (promoting fruit/vegetable consumption, health impacts of family meals and cooking, physical activity, food, and nutrition literacy) and collaborations with agencies/organizations partners (e.g., promoting and delivering nutrition education classes). The intent for this social media outreach and engagement strategy was to provide information and motivate healthy changes to eating behaviors—and to promote confidence in engaging a healthier diet, dietary behaviors, physical activity, and a healthier lifestyle for individuals, family, and children—in terms of cooking at home, food safety, and food resource management. Our posts varied with the seasons, reflecting the availability of foods, providing valuable health information, and offering helpful exercise tips. For instance, we tailored the posting schedule according to various U.S. holidays throughout the year, including New Year’s, Valentine’s Day, St. Patrick’s Day, Easter, Memorial Day, Independence Day, Labor Day, Halloween, Thanksgiving, and Christmas.

Each social media post included the HFCT SNAP-Ed website logo and tagline (“Spend Smart, Eat Well, Feel Great”). The Facebook and Instagram accounts are linked so that each post is shown on both platforms, allowing direct comparison of reach and engagement. Local community partners who serve low-income individuals and families regularly shared and marketed our Facebook and Instagram posts, amplifying our reach to low-income individuals. Although we did not conduct a preliminary analysis of the target audience’s preferences for images and colors, we relied on our knowledge of popular health education- and social marketing-related posts on Facebook and Instagram to help guide the development of design features including the selection of images and colors. The following were visual communication design elements: (1) images or static visuals include graphics or photos; (2) videos or moving pictures are grouped and presented in the form of snippets; and (3) reels or moving pictures are grouped in the form of snippets and usually shorter and more entertaining than conventional videos that are posted in a vertical layout. 

### 2.2. Procedure

We extracted data from our Facebook and Instagram posts shared between February 2021 and October 2022 (21 months). Post data were exported using Insights in Meta Business Suite. The following information was extracted for each post: publication dates, captions, reach, and engagement metrics (i.e., number of likes/reactions, comments, and shares). For Instagram, we also extracted the total number of saves for each post. We manually extracted reach and engagement metrics for reel videos on both platforms because the data were not available in the reports exported through Insights. In addition, we reviewed comments and excluded replies made by the HFCT team. Depending on the research objective, target audience, and platform used, user engagement could be defined and measured differently across studies [38,40]. For the analysis, total engagement included metrics presented on both social media platforms and was the sum of likes/reactions, comments, and shares. 

We did not record the incremental changes in the number of followers during the study period. On 31 October 2022 (the end of the study period), our Facebook and Instagram accounts had 495 and 1085 followers, respectively.

**Figure 1:**
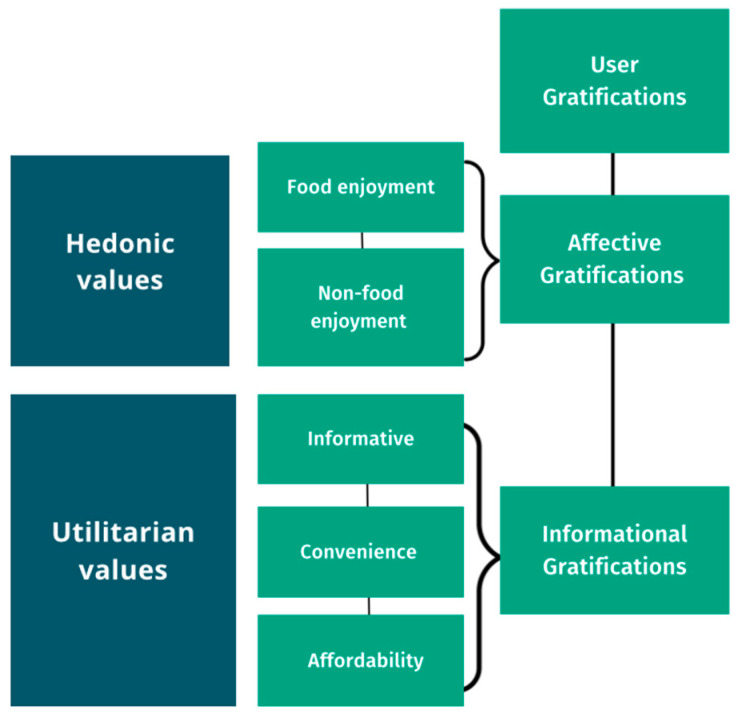
Two types of user gratifications as a framework for categorizing Healthy Family CT (HFCT) social media posts, according to hedonic values and utilitarian values.

We developed a theory-guided framework for testing the effectiveness of social media channels to reach and engage low-income audiences as part of implementation of the HFCT SNAP-Ed to promote healthier diets and activities for obesity prevention. This framework conceptualized user motivation for post reach and engagement via two major user gratification dimensions—affective (hedonic) and cognitive (utilitarian)—to categorize posts as reflecting hedonic and utilitarian values (Figure 1). This categorization scheme was developed based on prior studies that had considered the hedonic and utilitarian values category development [41,42].

**Figure 2:**
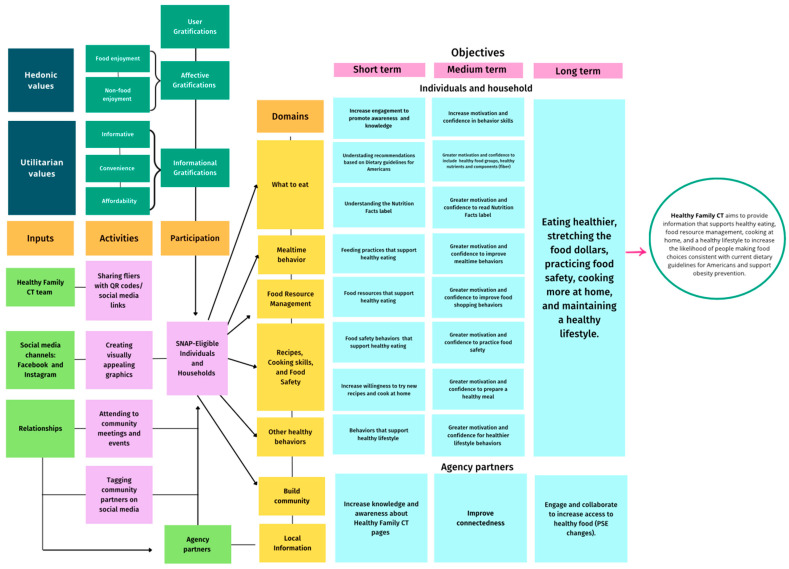
A framework for nutrition education dissemination that illustrates the user gratification (dark green) and nutrition education (yellow) categories, via a logic model framework with short-, medium-, and long-term outcomes.

The gratification categories were developed to tailor and improve the communication of the nutrition education categories. They were then incorporated in a logic model highlighting inputs from the HFCT team to promote the social media pages and activities of nutrition education outreach, and reach to low-income individuals and agency partners to achieve short-, medium-, and long-term nutrition education outcomes [43] (Figure 2). The nutrition education categories were constructed based on a SNAP-ED Toolkit and findings from previous research [44] and included the following: (1) *what to eat* (recommendations based on the 2020–2025 Dietary Guidelines); (2) *mealtime behaviors*; (3) *food resource management*; (4) *recipes*, including a highly consistent post layout, featuring close-up images of the dishes, cooking skills, and food safety; (5) *other healthy behaviors* (e.g., physical activity); and (6) *community building* (e.g., outreach activities). Our posts in multiple categories often highlighted local food-related information and resources (e.g., farmers markets, food pantries). We examined posts that included local information or resources as a subcategory in the framework to provide insights about locally accessible vs. more general nutrition information, as these findings can help inform SNAP-Ed social media strategies (i.e., more centralized/national vs local models of content development).

Our framework guided the conceptualization of the categories that we constructed through using the actual content of the posts included in the study period. We examined the data gathered from each post (including captions and links redirecting to the posts). The coding procedure was iterative and evolved as posts collected were systematically reviewed. D.C.A. developed the initial codebook with input from V.B.D., M.E.W., and C.A.L., who contributed to the contextualization of the content categories, resulting in an updated coding scheme. D.C.A. and V.B.D. carried out the coding tasks. Any discrepancies in coding among the researchers were discussed, and D.C.A. facilitated a resolution to resolve the coding discrepancies.

### 2.3. Data Analysis

For Aims 1 and 2, all descriptive analyses were performed using SPSS (version 28.0). We described the distributions of the engagement metrics with median value (IQR; range) as these metrics were quite skewed (typical of social media engagement data). Due to the low variability in separate engagement metrics (and overall low engagement per several of these metrics), total engagement was the sum of number of likes/reactions, replies, and shares. We compared engagement by post category on each social media platform (Facebook and Instagram). For the hedonic value category analysis, the hedonic groups combined taste- and food-based versus non-food (e.g., pleasurable activities such as yoga and gardening) categories. For the utilitarian value category, the groups included informative posts (e.g., dietary recommendations and guidance on using nutrition facts labels), convenience (e.g., easy-to-prepare recipes to boost culinary confidence), and affordability (e.g., making food shopping less expensive by choosing budget-friendly options). 

Reach and engagement variables were ranked using rank transformation (ties assigned to a low value) [45]. We used analysis of covariance (ANCOVA) to assess the main effects of post category on reach and engagement ranks, while controlling for the chronological posting from inception (coded from 1 to 453) to account for the incremental growth of social media presence, continually building new community partners, and the length of time a post was available for viewing and engagement. We also controlled for the frequency of reels within each category (yes/no), accounting for potentially higher reach and engagement with this visual communication design. The homogeneity of variances was assessed with Levene’s test. If equality of variances was not met, Quade’s non-parametric ANCOVA was used to test average differences between post categories. As this was an exploratory study, significant pairwise comparisons were also reported for a non-significant main effect of category. The statistical significance criterion was determined at the *p* < 0.05 level.

For Aim 3, we conducted a qualitative analysis of the features based on 15 posts that had the highest, moderate, and lowest engagement from the original set of posts (N = 442); these 15 posts were randomly selected, with 5 posts at each level of engagement. Two authors (D.C.A. and A.J.B.) independently examined the posts on Facebook or Instagram to identify features that differentiated low, moderate, and high levels of engagement. Only 2 posts in the moderate category were the same for Facebook and Instagram. 

Following previous research [46,47] and a review of the current study posts, the features coded for the current study included the image quality and composition (contrast, color balance), visual type (photo, graphic, and photo-graphic combo), and the total number of slides (for posts with multiple images). In addition, we also coded whether the post highlighted human presence (featuring community outreach or team member activities), used hashtags (indicating whether they were used and how many were included), and incorporating seasonal elements (including holidays, weather-related themes, seasonal events).

## 3. Results

### 3.1. Reach and Engagement by Platform and Change over Time

**Figure 3:**
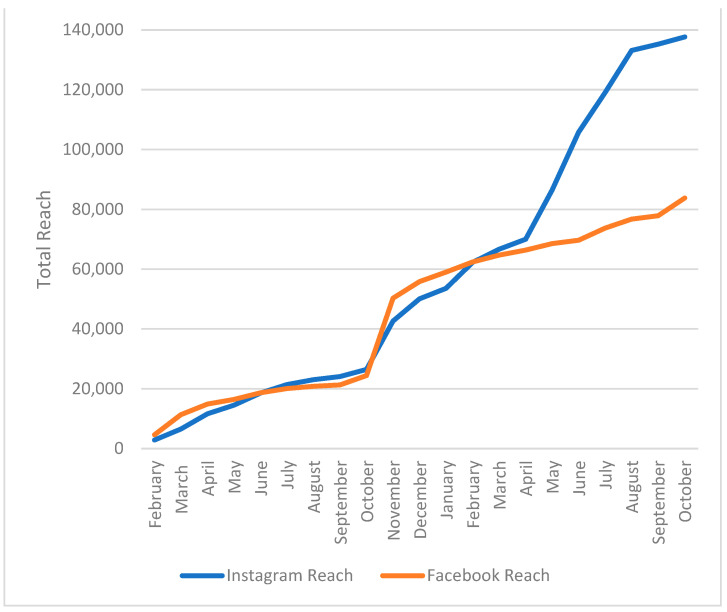
Additive organic reach (non-paid) of the Healthy Family CT (HFCT) by monthly interval on Facebook and Instagram between February 2021 and October 2022.

During the 21-month study period, the HFCT social media team published 453 original posts with a consistent number of posts per week (~5 posts/week) on both social media platforms; of these, 11 were reposts. All others were original organic posts (without being ‘boosted’ or paid), which generated a total reach of 83,275 unique users on Facebook and 136,621 on Instagram. Figure 3 shows the additive reach for each platform shown at monthly intervals. According to the demographic analysis of HFCT followers in October 2022, 91.4% of our Facebook and 80.4% of our Instagram followers were females. In terms of age, 63.5% of our Facebook and 36.5% of our Instagram followers were 45 years old or older.

**Figure 4:**
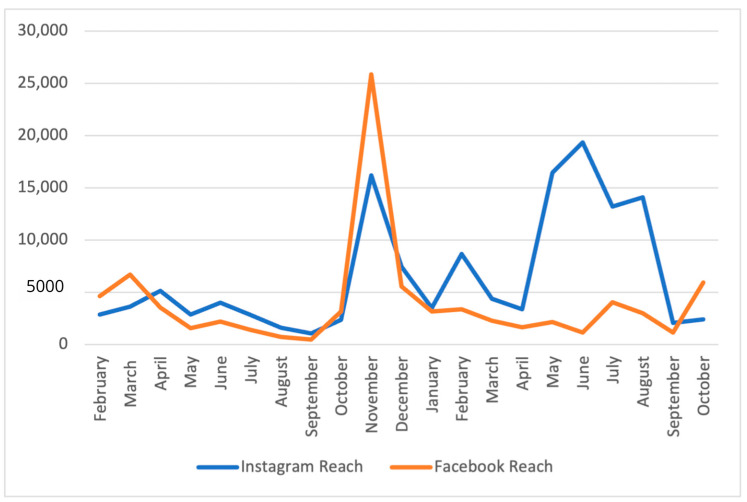
Monthly reach of the Healthy Family CT (HFCT) page Facebook and Instagram content from February 2021 to October 2022.

**Figure 5:**
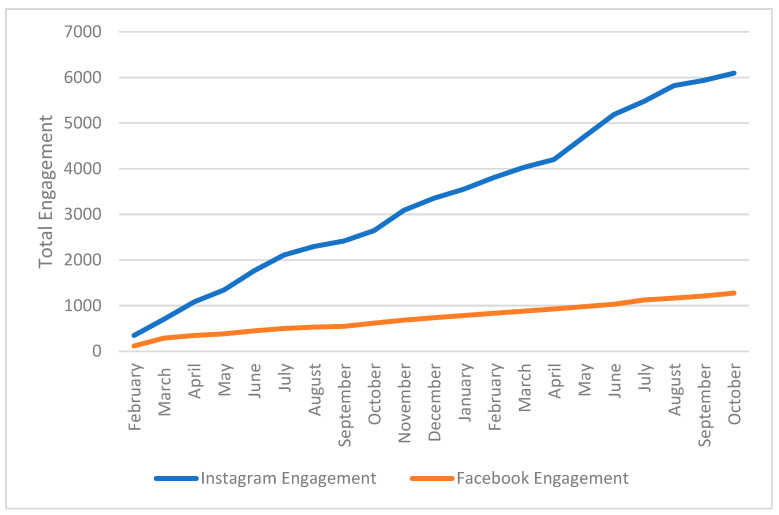
Additive organic, total engagement (sum of likes, users’ comments, and shares) of the Healthy Family CT (HFCT) page Facebook and Instagram content from February 2021 to October 2022.

Figure 4 shows the average monthly organic reach, highlighting the overall capacity of the HFCT content to reach Facebook and Instagram users based on our ~20 posts per month. Organic reach varied greatly depending on the social media platform and vacillated greatly from month to month. Facebook had a median organic reach of 43 unique users (IQR = 33–137, range: 17–21,732) per post and Instagram had a median organic reach of 107 unique users (IQR = 82.0–151.7, range = 17–14,333) per post. Facebook’s total monthly users ranged between 463 and 25,859 (median = 3005 users/month) and Instagram’s total monthly users ranged between 1064 and 19,248 (median = 3649 users/month). The most popular month for users on Facebook was November 2021, with more than 25,000 users reached; for Instagram, June 2022 was the most popular month, reaching nearly 20,000 users (Figure 5). These two different months with the unusually high reach (see the ‘spikes’) can be attributed to the two recipe posts that “went viral” during their respective period.

Figure 5 shows the total engagement (sum of comments, likes, and shares of a post) as additive growth each month, reaching a maximum of 6096 Instagram and 1276 Facebook users. The growth in total engagement for Instagram was fivefold during the study period. Instagram’s total monthly engagement frequency was between 115 and 480 (median 263/month), whereas Facebook’s total monthly engagement frequency ranged from 16 to 170 (median 50/month).

### 3.2. Reach and Engagement Frequency by User Gratification Categories

Table 1 describes the frequency of posts by gratification category as hedonic value (32% food and non-food enjoyment) and utilitarian value (36.6% informative, 19.7% convenience, and 11.7% affordability) on Facebook and Instagram by visual communication type (i.e., videos, images, and reels) as well as by reach and engagement metrics. For visual communication type, reels were most frequently found in the *convenience* subcategory of utilitarian value on Facebook and Instagram. These *convenience* posts also reached the most users on both platforms, including (1) 42,303 users on Facebook with a median (IQR) of 52 users (37–179) per post; (2) 71,892 users on Instagram with a median (IQR) of 123 users (87–165) per post.

### 3.3. Reach and Engagement Frequency by Nutrition Education Categories

The frequency of nutrition education posts on each social media channel according to reach, engagement metrics, and visual communication type is summarized in Table 2. Total reach varied widely depending on the visual communication type (i.e., videos, images, and reels). With 35,434 users (42.5%) on Facebook and 91,261 users (66.8%) on Instagram reached by reels, more users were reached with reels than videos and images. Although reels tended to have greater reach, images were the main type of visual communication presented on both platforms (around 82% of all posts).

The most popular content was *recipe* posts (n = 156, 35.3%). These posts reached 51,251 users on Facebook with a median (IQR) of 50 (35–159, range = 24–21,732) per post; they also reached 93,536 users on Instagram with a median of 109 (87–160, range = 17–14,333) per post. On Facebook, posts promoting *local-food-related information* had higher engagement (median [IQR] = 3, range = 1–5 likes). Instagram posts promoting *mealtime behaviors* had a lower engagement level (median [IQR] = 5, range 4–7 likes) than *local food-related information* and *food resources management* posts did, which had a higher engagement level (median [IQR] = 12, range 9–18 likes); median [IQR] = 9, range 7–14 likes), respectively).

**Figure 6:**
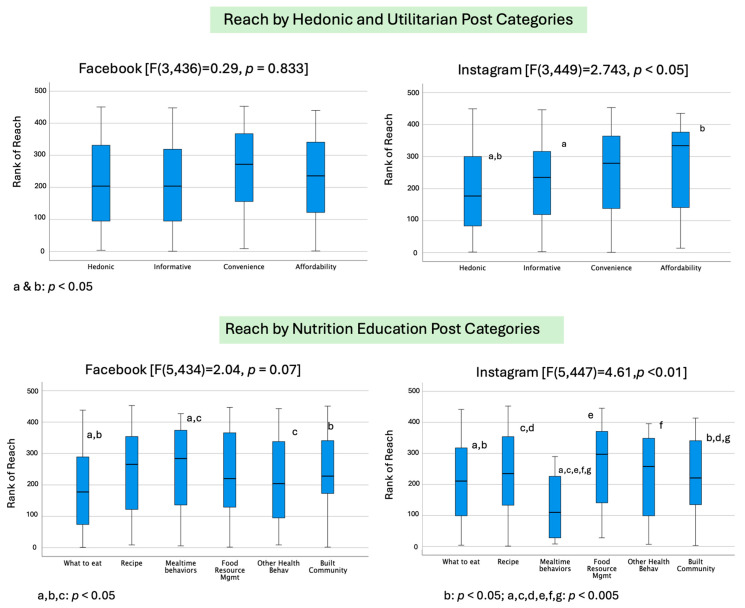
Reach in relation to communication approach and nutrition education categories: hedonic and utilitarian value posts on Facebook (top left) and Instagram (top right) and nutrition education posts on Facebook (bottom left) and Instagram (bottom right). Superscripts of the same letter represent statistically significantly differences in pairwise comparison with *p*-values for each pairwise comparison as shown.

Controlling for the chronological order of posts and frequency of reels, no difference was found for Facebook reach between hedonic and utilitarian values (F(3,436) = 0.29, *p* = 0.833; Figure 6). For Instagram, there was a statistically significant difference for reach (F(3,449) = 2.743, *p* < 0.05). Specifically, utilitarian posts including *affordability* (*p* = 0.042) and *informative* posts (*p* = 0.012) had significantly higher average reach than hedonic posts.

**Figure 7:**
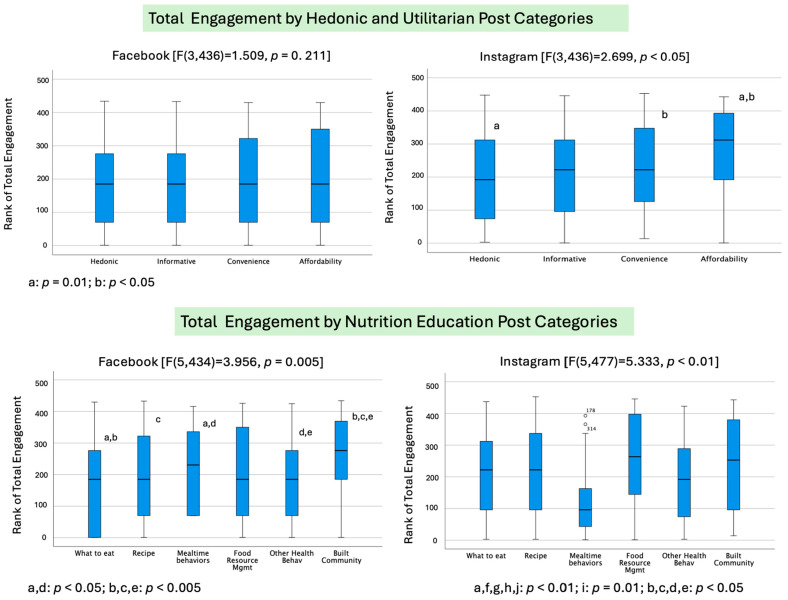
Total engagement in relation to communication approach and nutrition education categories: hedonic and utilitarian value posts on Facebook (top left) and Instagram (top right) and nutrition education posts on Facebook (bottom left) and Instagram (bottom right). Superscripts of the same letter represent statistically significantly differences in pairwise comparison with *p*-values for each pairwise comparison as shown.

For the nutrition education categories, by controlling for the chronological order of posts and frequency of reels, there was a trend for an overall effect on Facebook reach (F(5,434) =2.04, *p* = 0.07); Figure 7); *mealtime behaviors* (*p* = 0.024) and/or *community building* (*p* = 0.019) had greater reach than *what to eat* posts. For Instagram, there were significant main effects on reach (F(5,447) = 4.61, *p* < 0.001), with the lowest reach associated with *mealtime behaviors* from any other categories and higher reach associated with *community building*, when compared to *what to eat* (*p* = 0.021) and *recipes* (*p* < 0.001).

**Figure 8:**
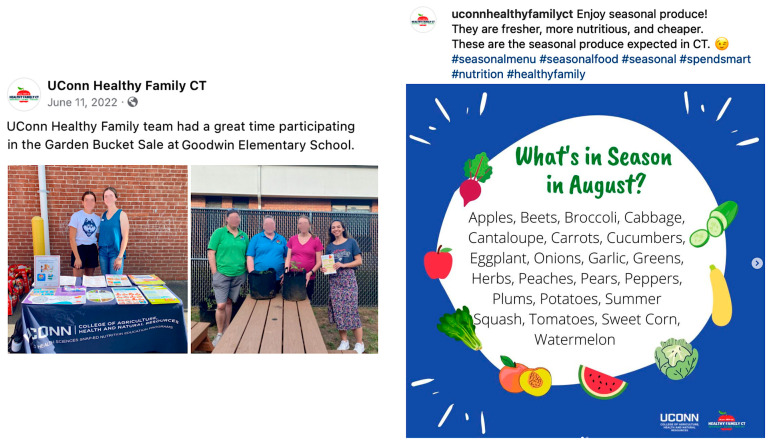
Sample posts with highest engagement on Facebook and Instagram.

The average differences in total engagement on Facebook and Instagram showed similar patterns via total reach for the hedonic and utilitarian values and nutrition education categories (Figure 8). For Instagram, there was a significant overall effect on engagement (F(3,436) = 2.699, *p* = 0.045) regarding gratification categories. Specifically, the *affordability* category (utilitarian post) had a significantly higher average engagement than the *hedonic* (*p* = 0.01) and *convenience* categories (*p* = 0.015) on Instagram. We also found a significant overall effect of engagement on Facebook (F(5,434) = 3.956, *p* = 0.005) and Instagram (F(5,477) = 5.333, *p* < 0.001) for the nutrition education categories. For Facebook, the *community building* category had significantly greater engagement than most other categories, while *what to eat* generated the lowest engagement compared to *community building* (*p* < 0.001) and *mealtime* (*p* = 0.038). For Instagram, *mealtime behaviors* posts elicited significantly lower engagement compared to *what to eat* (*p* = 0.006), *recipes* (*p* = 0.047), *food resource management* (*p* = 0.002), and *community building* (*p* < 0.001) posts.

**Figure 9:**
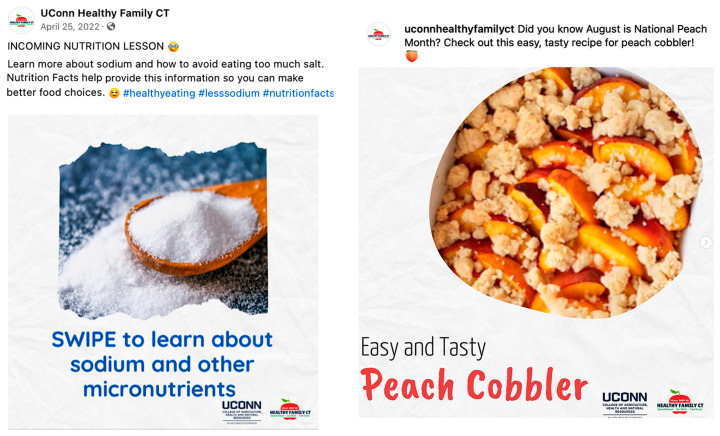
Sample social media posts with lowest engagement on Facebook and Instagram.

Among the 15 randomly selected posts reflecting low, medium, and high engagement for qualitative analysis, it appeared that high-engagement posts were more likely to include real people in them. Similarly, posts incorporating seasonal elements also received higher engagement during relevant periods, including announcements of seasonal produce, holidays, national celebrations, and awareness months (e.g., black history month) (Figure 8). While we did not perform a qualitative comparison based on color selections, low-engagement posts appeared more likely to present low-color-contrast or less-appealing images, particularly on Instagram (Figure 9).

## 4. Discussion

The current study, guided by a theoretical framework, examined Facebook and Instagram user reach and engagement elicited by evidenced-based nutrition education that aimed to promote healthy diet, overall health, food resource management, and access to healthy and affordable food. The target audience of low-income adults and families was reached organically, i.e., not via paid or promoted posts. Instagram outpaced Facebook in total reach and engagement from February 2021 to October 2022. We also found that reach and engagement on Instagram differed by categories capturing user gratification as reflected in hedonic versus utilitarian values and nutrition education topics.

For Instagram, *affordability* presented higher reach and engagement. While *mealtime behaviors* had the lowest reach and engagement on Instagram, this category achieved greater reach and engagement on Facebook. Posts related to dietary guidance, food resource management, and connection with local community resources elicited greater reach and engagement on both platforms. Our results supported the effectiveness of utilizing both Facebook and Instagram to conduct communication outreach and engagement to low-income adults and families interested in learning and benefiting from the nutrition education information, framed with a simple narrative of living an easily achievable healthy and active lifestyle.

The findings from this study also evidence the importance of engaging the target audience and community stakeholders with locally relevant and tailored social media content on Facebook and Instagram. The *community building* category of our posts that highlighted social connectedness had the highest user reach and engagement levels among most nutrition education categories. This type of content helps promote authenticity, a personal connection with the audience, and increased audience engagement [48].

The *Healthy Family CT* (HFCT) team has actively posted original, non-paid content and built relationships with local community and government agency partners (e.g., nutrition education outreach) to provide opportunities for better access to nutrition information to target audiences. Local partnerships on social media have been described in an exploratory study as effective and economical to engage the low-income public [49]. For example, partnerships with local agencies (e.g., federal and state-designated non-profits working with the low-income population) can include tagging and cross-posting content to inform and engage low-income individuals with useful food- and health-related resources on social media platforms [49]. These strategies are consistent with the recommendations to improve the overall sense of community, as suggested by the authors of a previous study of a University Extension Facebook page [50]. In our study, it appeared that posts that included local-food-related resources had higher median likes on both Facebook and Instagram than other post categories, which may indicate that people viewing these posts found the content relevant or helpful. These findings suggest that while developing SNAP-Ed social media content at a national or state level may be an efficient use of resources, having local groups post resources may be an appealing and effective strategy to reach and engage low-income families in their community.

According to the HFCT social media page followers’ demographics, our Instagram presence has a younger audience (65% were 44 years old or younger) relative to Facebook (37% were 44 years old or younger). This finding matches trends in social media platform use by US adults [51] and US parents [52]. Different social media platforms may attract different sub-populations. Understanding which social media platforms are used by the target population—and why and how they use the platform—is critical for developing and disseminating evidence-based and engaging tailored health promotion content.

Previous research has also indicated that Facebook is an effective venue to engage low-income families [6,7,8]. To our knowledge, Instagram’s effectiveness as a nutrition education tool has yet to be fully explored. The current study has hence expanded on this limited body of literature and contributed to enhancing our understanding of how social media platforms may be utilized to reach and engage low-income communities with easy-to-master nutrition information for making sound food and lifestyle choices.

Although eating pleasure may encourage healthy eating [42], the reach and engagement levels for posts promoting food enjoyment were not significant when compared to the utilitarian categories. Instead, our study found that *affordability* posts had a greater reach on Instagram and larger total engagement on both social media platforms. These findings suggest that our attitude toward food is not necessarily defined by either hedonic or utilitarian value alone. This observation is consistent with a study that examined how users communicated about food online, which concluded that while the concept of *dinner* could be associated with hedonic value for some people, it may also fulfill a utilitarian need for others [41]. For instance, the authors pointed out that when discussing food shopping and specific locations for finding products with the best value, a utilitarian approach to food consumption emerged [41].

Our study considered the attitude toward food preparation an opportunity to promote content that can encourage cooking more at home to achieve affordability and healthy eating and to maximize the utilitarian value of food consumption. As such, our findings add to the growing evidence that social media is an effective and promising tool for promoting healthy eating, particularly addressing low-income populations’ needs, such as eating healthy on a budget [7,8,53].

Diet quality is often associated with home cooking and food preparation in the nutrition literature [54]. In the present study, posts promoting *recipes* that covered cooking skills and food safety were the most frequently accessed in the nutrition education category and generated the highest reach on Instagram. These findings are similar to past work that reported how recipe posts were the most popular food content searched on Instagram [55]. Consistent with our findings, an intervention assessing the impact of a Cooking Matters nutrition education Facebook page on directing low-income parents to improve healthy eating behaviors showed that as *recipes* received the most likes, a significant improvement emerged between pre- and post-outcomes associated with feeding children healthy meals within the budget [7].

Our study also found a significant engagement on both platforms for *food resource management* posts, highlighting target audience interest in learning fundamental skills—such as meal planning and food shopping behaviors—for supporting healthy eating and affordable food choices. In the present study, posts about *mealtime behaviors* had significantly greater user reach on Facebook than on Instagram. These findings also agree with those of a previous study, which evaluated mealtime posts as the value of parents eating with children and found this type of post content to be the third most viewed on a Facebook page [6]. As Instagram had the lowest user reach and engagement levels in the nutrition education category, this suggests that our audiences on Facebook and Instagram find separate topics to be more relevant and engaging. These results support that researchers, public health professionals, and community organizations who intend to promote healthy eating should tailor their social media feeds to the differential interests of their audiences.

Our qualitative analysis found that high-engagement posts were more likely to feature team members, community outreach activities, or seasonal content compared to posts with lower engagement. By implication, our findings again highlight the importance of promoting outreach activities to foster community engagement, as visual images featuring human faces garnered more user engagement on both social media platforms and aligned with previous research findings [47,56]. However, conflicting results exist regarding which platform performs better with this strategy [47], as well as the implementation in the context of public health [57]. Still, longitudinal data are needed to understand the relationship between social media use and food- and nutrition-related health outcomes. Additionally, when creating content for social media, it is important to consider image quality and composition to ensure visually appealing final products [58], especially on Instagram. This is because Instagram is a more casual and detached form of posting [59], where the picture’s quality and colorfulness seem to be more important [59,60]. Its visual format may also facilitate comprehension for users with lower educational attainment [61]. As health promotion practitioners engage with diverse audiences through social media, incorporating visuals into institution-generated content can help drive engagement on Facebook and Instagram [62].

### Strengths and Limitations

Our study has the following limitations. First, the U&G literature considers social media platform such as Facebook a virtual space for users to enjoy building/joining a user community and deriving a sense of social identity [26,38]. The current study operationalized *community building* as a nutrition education category, as our social media posts highlighted nutrition education outreach. Future research should also consider the posts that aim to help build a community of users/followers—around making healthy food choices and mastering the nutritional tips—as a user gratification category affiliated with a utilitarian value.

Second, the study team did not track the changes in the number of followers over time. Although we have the aggregate demographic information (e.g., age and gender) about users who follow our social media pages, we do not have access to the demographics of individual users who interacted with each post. Additional studies should track the demographic data associated with users who interacted with the nutrition education content under study here to enable more user-specific analysis of post access frequency and engagement. Third, we utilized the free version of Meta Business Suite to track and identify user interactions with our social media posts. For this reason, there were also some limitations regarding analytics programs and metrics that were not available or were under development (at the time of data collection), which restricted our ability to potentially report selected additional findings of interest.

## 5. Conclusions

In this exploratory analysis, we found that Facebook and Instagram posts promoting evidence-based nutrition education are effective communication channels—to reach and engage users through unpaid amplification of the posts as well as low-income individuals and families—in collaboration with local community partners. Our study also contributes to exhibiting the benefits of adopting a theory-guided approach to examine and analyze communication strategies that target nutrition education dissemination with the goal of increasing message reach to and engagement of low-income families who are social media users.

In sum, maximal user reach and engagement varied by platform and by category of post, whether analyzed based on the use and gratifications theory or nutrition education topics. Thus, nutrition and health educators should focus on developing social media-based outreach strategies whose content is strategically tailored for their target audiences and the social media platforms they use. To achieve an optimal level of engagement, it is also essential to consider the power of building community involvement and sharing timely content (e.g., seasonal posts) when developing social media engagement strategies to achieve greater impact. The current study also provides a valid and valuable example of how best to utilize social media use reach and engagement data to better understand the nutrition education content that may generate the most significant dissemination and engagement and for creating the potentially strongest impact on eating and public health. It is recommended that researchers and nutrition educators who plan to develop and disseminate social media content be encouraged to take a similar iterative, data-driven approach to content development.

## Figures and Tables

**Table 1:** Reach and engagement metrics by hedonic and utilitarian value categories.

Characteristic	All Posts (*n* = 442)	Hedonic (*n* = 141, 32%)	Informative (*n* = 162, 36.6%)	Convenience (*n* = 87, 19.7%)	Affordability (*n* = 52, 11.7%)
**Facebook**					
**Visual communication n (%)** posts					
Video posts	61 (13.8)	18 (12.8)	11 (6.8)	20 (23.0)	12 (23.1)
Image posts	362 (81.9)	121 (85.8)	147 (90.7)	55 (63.2)	39 (75.0)
Reels (short video) posts	19 (4.3)	2 (1.4)	4 (2.5)	12 (13.8)	1 (1.9)
**Organic Reach, median (IQR; range)**	43 (33–137; 17–21,732)	41 (32–132; 20–1632)	41 (33–96; 17–1231)	52 (37–179; 24–21,732)	44 (34–145; 19–751)
**Engagement, median** **(IQR; range)**					
Likes	2 (1–3; 0–369)	2 (1–3; 0–38)	1 (1–3; 0–13)	2 (1–4; 0–369)	2 (1–4; 0–12)
Replies	0 (0–0; 0–9)	0 (0–0; 0–4)	0 (0–0; 0–3)	0 (0–0; 0–9)	0 (0–0; 0–1)
Shares	0 (0–1; 0–108)	0 (0–1; 0–9)	0 (0–1; 0–6)	0 (0–1; 0–108)	0 (0–1; 0–6)
**Instagram**					
**Visual communication n (%)** posts					
Video posts	35 (7.9)	7 (5.0)	7 (4.3)	16 (18.4)	5 (9.6)
Image posts	365 (82.6)	123 (87.2)	147 (90.7)	55 (63.2)	40 (76.9)
Reels (short video) posts	42 (9.5)	11 (7.8)	8 (4.9)	16 (18.4)	7 (13.5)
**Organic Reach, median (IQR; range)**	107 (82–152; 17–14,333)	95 (76–134; 26–6330)	110 (83–141; 42–4267)	123 (87–165; 17–14,333)	150 (88–174; 56–838)
**Engagement, median users (IQR; range)**					
Likes	9 (5–13; 0–271)	8 (5–12; 1–80)	8 (5–12; 0–41)	9 (6–15; 2–271)	11 (7–16; 0–32)
User comments	0 (0–0; 0–4)	0 (0–0; 0–4)	0 (0–0; 0–1)	0 (0–0; 0–2)	0 (0–1; 0–3)
Shares	0 (0–2; 0–57)	0 (0–2; 0–9)	0 (0–2; 0–28)	0 (0–2; 0–57)	1 (0–3; 0–16)
Saves of the content	0 (0–1; 0–206)	0 (0–1; 0–44)	0 (0–1; 0–8)	1 (0–2-0–206)	0 (0–1; 0–5)

**Table 2:** Reach and engagement metrics by nutrition education category.

Characteristic	What to Eat *(n* = 125) 28.3%	Recipes (*n* = 156) 35.3%	Mealtime Behaviors (*n* = 24) 5.4%	Food Resources Management (*n* = 43) 9.7%	Healthy Behaviors (*n* = 53) 12%	Community Building (*n* = 41) 9.3%	Local Information (*n* = 29) 6.6%
**Facebook**							
**Visual communication n (%)**							
Video posts	12 (9.6)	30 (19.2)	0	8 (18.6)	10 (18.9)	1 (2.4)	4 (13.8)
Image posts	111 (88.8)	114 (73.1)	23 (95.8)	32 (74.4)	42 (79.2)	40 (97.6)	24 (82.8)
Reels (short video) posts	2 (1.6)	12 (7.7)	1 (4.2)	3 (7.0)	1 (1.9)	0	1 (3.4)
**Organic Reach, median users** **(IQR; range)**	38 (31–66; 17–696)	50 (35–159; 24–21,732)	58 (35–195; 23–534)	44 (35–180; 19–1152)	41 (30–128; 24–974)	42 (35–104; 19–1632	52 (40–138; 24–1632)
**Engagement,** **median users** **(IQR; range)**							
Likes	1 (0–2; 0–12)	1 (1–3; 0–369)	2 (1–3; 0–6)	2 (1–4; 0–10)	1 (1–2; 0–8)	2 (1–4; 0–38)	3 (1–5; 0–38)
User Comments	0 (0–0; 0–3)	0 (0–0; 0–9)	0 (0–0; 0–4)	0 (0–0; 0–1)	0 (0–0; 0–1)	0 (0–0; 0–2)	0 (0–0; 0–2)
Shares	0 (0–1; 0–6)	0 (0–1; 0–108)	0 (0–1; 0–3)	0 (0–1; 0–4)	0 (0–1; 0–6)	0 (0–1; 0–9)	0 (0–1; 0–2)
**Instagram**							
**Visual communication n (%)**							
Video posts	3 (2.4)	20 (12.8)	0	1 (2.3)	10 (18.9)	1 (2.4)	0
Image posts	112 (89.6)	116 (74.4)	23 (95.8)	32 (74.4)	42 (79.2)	40 (97.6)	26 (89.7)
Reels (short video) posts	10 (8.0)	20 (12.8)	1 (4.2)	10 (23.3)	1 (1.9)	0	3 (10.3)
**Organic Reach** **median users** **(IQR; range)**	106 (79–142; 46–1436)	109 (87–160; 17–14,333)	80 (60–107; 51–128)	135 (88–170; 60–4267)	116 (79–156; 49–190)	104 (87–152; 42–231)	134 (94–178; 42–6330
**Engagement,** **median users** **(IQR; range)**							
Likes	8 (5–13; 1–28)	10 (6–14; 1–271)	5 (4–7; 0–19)	9 (7–14; 0–41)	7 (5–11; 1–19)	9 (6–14; 2–32)	12 (9–18; 2–42)
User Comments	0 (0–0; 0–4)	0 (0–0; 0–3)	0 (0–0; 0–2)	0 (0–0; 0–3)	0 (0–0; 0–2)	0 (0–0; 0–1)	0 (0–0; 0–1)
Shares	0 (0–2; 0–16)	0 (0–2; 0–57)	0 (0–1; 0–4)	2 (0–3; 0–16)	0 (0–2; 0–6)	0 (0–2; 0–22)	2 (0–2; 0–28)
Saves of the content	0 (0–1; 0–6)	0 (0–1; 0–206)	0 (0–1; 0–1)	1 (0–2; 0–8)	0 (0–0; 0–4)	0 (0–0; 0–2)	0 (0–1; 0–7)

## Data Availability

The original contributions presented in the study are included in the article, further inquiries can be directed to the corresponding author.
